# Influence of the Preparation Method on the Structural, Morphological and Dielectric Properties of FeNbO_4_ Ceramics

**DOI:** 10.3390/ma16083202

**Published:** 2023-04-18

**Authors:** Susana Devesa, Filipa Gonçalves, Manuel Graça

**Affiliations:** 1 CFisUC, Physics Department, University of Coimbra, Rua Larga, 3004-516 Coimbra, Portugal; 2 I3N and Physics Department, University of Aveiro, 3810-193 Aveiro, Portugal

**Keywords:** FeNbO_4_, colloidal gel, polymeric gel, rietveld refinement, radiofrequency, microwaves

## Abstract

In this work, iron niobate (FeNbO_4_) was prepared via two processes based on the sol-gel method: colloidal gel and polymeric gel. The obtained powders were submitted to heat treatments at different temperatures based on the results obtained via differential thermal analysis. The structures of the prepared samples were characterized via X-ray diffraction and the morphology was characterized via scanning electron microscopy. The dielectric measurements were performed in the radiofrequency region using the impedance spectroscopy technique and in the microwave range using the resonant cavity method. The influence of the preparation method was noticeable in the structural, morphological and dielectric properties of the studied samples. The polymeric gel method promoted the formation of monoclinic and orthorhombic iron niobate at lower temperatures. The differences in the samples’ morphology were also remarkable, both in the size and shape of the grains. The dielectric characterization revealed that the dielectric constant and the dielectric losses had the same order of magnitude and similar trends. A relaxation mechanism was identified in all the samples.

## 1. Introduction

In recent decades, the interest in the research of ceramic materials based on lead-free metal oxides has grown due to their excellent catalytic, chemical, magnetic and electrical properties. Among the most studied oxides are the perovskites ABO_3_ (where A = Li, Na, La, etc., and B = Nb, Fe, etc.) and the mixed metallic oxides ABO_4_ (where A, B = Bi, V, Zn, Ce, Co, Fe, Nb, etc.), which are prepared using different physical and chemical methods, due to their potential applicability in numerous fields, namely, in gas sensor, catalytic and photodetector technologies [1,2,3,4,5,6,7,8].

From these, one of the well-known mixed metallic oxides is the iron niobate, FeNbO_4_, which possesses both magnetic and electric properties and shows potential in a wide variety of applications. Among these applications, solar energy conversion, gas sensors, catalysts, photoanode material and a precursor in the preparation of single-phase Pb(Fe_0.5_Nb_0.5_)O_3_ are highlighted in the literature [2,3,9].

**Figure 1:**
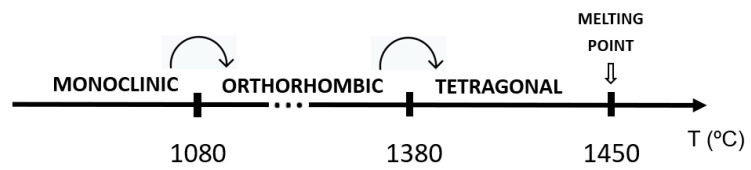
Temperature evolution of the FeNbO_4_ polymorphs (adapted from [2]).

Iron niobates exhibit three different crystalline phases depending on the annealing temperature: a monoclinic phase (wolframite type, space group P2/c), which forms at a temperature below 1080 °C; an orthorhombic phase of α-PbO_2_-type (space group Pbcn), which can be obtained at 1080 °C ≤ T ≤ 1380 °C; and the tetragonal rutile phase (space group P4_2_/mnm), which is formed above T = 1380 °C and below the melting point (T ≈ 1450 °C), as shown in Figure 1 [2,8,9,10]. A further monoclinic structure, which crystallizes in the GaNbO_4_ type (space group C2) was reported by Brunner and Gruehn [11] and was obtained via the gas transport preparation method in the temperature range of 600–900 °C [8].

**Figure 2:**
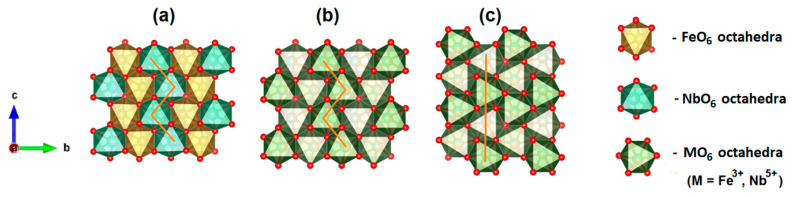
Schematic illustrations of crystal structures of FeNbO_4_ (drawn using VESTA software, version 3): (**a**) monoclinic; (**b**) orthorhombic; (**c**) tetragonal.

Figure 2 shows the three main polymorphs of the FeNbO_4_ compound. The monoclinic structure is based on the zig-zag arrangement along the c-axis of FeO_6_ and NbO_6_ octahedra. These chains of octahedrons are connected by sharing the corner oxygen atoms along the a- and b-axes. The orthorhombic structure is similar; however, the iron and niobium atoms appear randomly distributed. The tetragonal structure is identical to the orthorhombic structure, yet the arrangement of octahedrons occurs linearly [8,9].

Over recent decades, several studies were presented to improve the synthesis of FeNbO_4_, either through the optimization of the starting materials or through the optimization of the processing method. The ultimate goal of ceramic processing is to achieve the desired phase while keeping the sequence of steps as simple as possible. As is recognized, both the starting materials and the processing history critically affect the properties of the final product, and thus, it is crucial to evaluate the different processing techniques [6,12]. Considering that purity and reactivity are key factors, it is also necessary to contemplate the characteristics of the crystal phases formed and the relationships between the method of preparation and the properties of the obtained product [12].

In 1996, Tena et al. prepared FeNbO_4_ using three different methods: the solid-state reaction method and two processes based on the sol-gel method. In the solid-state reaction method, Nb_2_O_5_ and Fe_2_O_3_ were used as precursors, which were mixed in stoichiometric amounts and homogenized in acetone in a planetary ball mill for 20 min. In the case of the processes based on the sol-gel method, two types of gel were produced: colloidal and polymeric. For the preparation of the colloidal gel, NbCl_5_ and FeCl_3_·6H_2_O were used, while in the polymeric gel route, NbCl_5_ and FeCl_3_·6H_2_O were the precursors. To obtain the colloidal gel samples, NbCl_5_ was dispersed in water via vigorous stirring. Then, FeCl_3_·6H_2_O was added and the mixture was stirred vigorously at 70 °C. Thereafter, an NH_4_OH solution was added until gelation occurred (pH = 5–6). Finally, the gel obtained was dried with an infrared lamp. In the polymeric gel method, a solution of NbCl_5_ dissolved in ethanol was subjected to continuous stirring at 70 °C for 15 h. FeCl_3_·6H_2_O was added, keeping the mixture at 70 °C for 24 h, and finally acidified with HNO_3_. The samples were thermally treated at temperatures between 300 and 1000 °C for 12 h. According to the authors, the presence of FeNbO_4_ with an orthorhombic structure as the only crystal phase formed was detected in samples treated at 1000 °C. However, there is no information about which of the methods produced this single-phase sample. Besides this, the authors did not present the results of the structural characterization performed [13].

In 1999, Ananta et al. synthesized FeNbO_4_ via the solid-state reaction method, using Fe_2_O_3_ and Nb_2_O_5_ as precursors. The samples obtained were calcined at temperatures between 1075 and 1150 °C over periods between 2 and 5 h. This study showed that the Fe_2_O_3_ phase coexisted with the monoclinic FeNbO_4_ phase for treatment temperatures below 1100 °C. By increasing the calcination temperature to 1150 °C, the monoclinic FeNbO_4_ completely converted to an orthorhombic structure [6].

In 2002, Theissmann et al. presented the synthesis of FeNbO_4_ via the sol-gel method using NbCl_5_ and FeCl_3_·6H_2_O as precursors. In the sample treated at 1050 °C for 12 h, pure monoclinic FeNbO_4_ was identified. The authors pointed out that, contrary to what was verified in the previous works cited by them, the secondary phase FeNb_2_O_6_ was not identified [14].

In 2008, Cho et al. prepared FeNbO_4_ via the solid-state reaction method and a hydrothermal method. In the case of the solid-state reaction method, Fe_2_O_3_ and Nb_2_O_5_ were used as precursors, with the sample being treated at 1000 °C. For the hydrothermal method, the precursors Fe(NO_3_)_2_·6H_2_O and NbCl_5_ were dissolved separately in water and ethanol, respectively, under constant magnetic stirring. To the obtained niobium ethoxide solution was added an aqueous iron nitrate solution. The pH of the solution was adjusted using NH_4_OH. The mixture was placed in an autoclave, heated to 180 °C and maintained for 12 h with stirring, followed by natural cooling to room temperature. The reddish-brown precipitate obtained was collected by centrifugation and washed with distilled water and ethanol. After drying at 90 °C in an oven, the powder was heat treated at 600, 800 and 1000 °C. In the sample prepared via the solid-state method, monoclinic FeNbO_4_ was identified after a heat treatment at 1000 °C, while via the hydrothermal method and a heat treatment of 600 °C, the authors claimed to have obtained pure orthorhombic FeNbO_4_ [15].

In 2014, Babu et al. prepared FeNbO_4_ via the hydrothermal method using Fe(NO_3_)_3_·9H_2_O and NbCl_5_ as precursors. The authors followed the procedure proposed by Cho et al., which is described above. Thermal treatments were carried out at 600 and 800 °C and the authors claimed to have obtained pure orthorhombic FeNbO_4_ in both samples; however, at 600 °C, there was still amorphous material present [16].

Again in 2014, Li et al. prepared FeNbO_4_ via the solid-state reaction method using Fe_2_O_3_ and Nb_2_O_5_ as precursors. The samples were subjected to two heat treatments at 1000 °C for 24 h and only monoclinic FeNbO_4_ was identified [17].

Devesa et al. synthesized FeNbO_4_ in 2016 via the sol-gel method using NbCl_5_ and Fe(NO_3_)_3_·9H_2_O as precursors. The samples were thermally treated at temperatures between 500 and 1200 °C for 4 h. In the samples treated between 500 and 1000 °C, monoclinic FeNbO_4_ was identified, as well as an additional phase of Fe_2_O_3_ with content equal to or less than 5%. For the heat treatment of 1200 °C, a single-phase sample of monoclinic FeNbO_4_ was obtained [18].

In 2019, Ahmed et al. prepared FeNbO_4_ by applying several methods, such as the co-precipitation method, the surfactant-assisted co-precipitation method, the hydrothermal method and the sol-gel method. The co-precipitation method employed Fe(NO_3_)_3_·9H_2_O and C_4_H_4_NNbO_9_·xH_2_O as precursors. Using NH_4_OH, the pH of the solutions was adjusted to 2, 7 and 10. In the surfactant-assisted co-precipitation method, ethylene glycol, polyethylene-400 and sodium dodecyl sulfate were added to the precursors utilized in the previously described process. In the hydrothermal method, the solutions formed by the precursors with the pH adjusted to the same values were subjected to heating in an autoclave for 24 h. Finally, in the sol-gel method, the precursors C_6_H_5_FeO_7_ and C_4_H_4_NNbO_9_·xH_2_O were used. In addition to the solution initially obtained with a pH equal to 2, solutions with pHs of 7 and 10 were prepared using NH_4_OH for this adjustment. Regardless of the preparation method, the obtained powders were thermally treated at 1100 °C for 6 h. In the samples prepared via co-precipitation, in addition to monoclinic FeNbO_4_, the phases Fe_2_O_3_ and NbO_2_ were identified. The samples prepared via the surfactant-assisted co-precipitation method had two phases: monoclinic FeNbO_4_ and NbO_2_. In the samples prepared via the hydrothermal method and the sol-gel method, the monoclinic FeNbO_4_ and the secondary phase Fe_2_O_3_ were identified [2].

In 2019, Lakshminarasimhan et al. synthesized FeNbO_4_ via the solid-state reaction method using Fe_2_O_3_ and Nb_2_O_5_ as precursors. The powder produced was calcined at 750 °C for 12 h and then at 1200 °C for 24 h. In the sample removed from the oven and placed in contact with air, pure-phase orthorhombic FeNbO_4_ was identified. In the case of the sample whose cooling took place inside the oven, according to its inertia, the pure phase of monoclinic FeNbO_4_ was identified [19].

In 2020, Liu et al. prepared FeNbO_4_ via the solid-state method using Fe_2_O_3_ and Nb_2_O_5_ as precursors. The pure monoclinic phase was formed after thermal treatment at 1050 °C for 5 h, with cooling occurring at a rate of 5 °C/min. Reheating the sample at 1200 °C for 0.5 h, followed by cooling in air, promoted the formation of orthorhombic FeNbO_4_ [20].

In 2021, Bezerra et al. carried out the synthesis of FeNbO_4_ via the solid-state reaction method with Fe_2_O_3_ and Nb_2_O_3_ as precursors. The obtained powders were thermally treated at 1200 °C for 4 h, obtaining monoclinic FeNbO_4_ [21].

Despite the several studies devoted to the production and characterization of iron niobate, it is still challenging to synthesize a single-phase m-FeNbO_4_ and/or o-FeNbO_4_ ceramic, with the authors reporting different secondary phases. Besides the precursors and amorphous material, FeNb_2_O_6_ and/or Fe_4_Nb_2_O_9_ can be regularly formed [6,14]. Ananta et al. [6] also declared that over a wide range of calcination conditions, the single-phase m-FeNbO_4_ cannot be produced easily. This statement can be corroborated by the analysis of the mentioned works. Besides this, contradictory information can be found, namely, considering that there are works that announced the formation of single-phase o-FeNbO_4_ at low treatment temperatures of 600 and 800 °C [15,16] when the transition from m-FeNbO_4_ to o-FeNbO_4_ is expected at temperatures close to 1080 °C [2,8,9,10].

For the reasons mentioned above and the importance of the synthesis process and starting materials in the final product, FeNbO_4_ synthesis requires more investigation. 

In this work, FeNbO_4_ samples were prepared using two processes based on the sol-gel method, namely, the colloidal gel method and the polymeric gel method, and were heat-treated at temperatures between 400 °C and 1200 °C. The structural and morphological studies were accomplished via X-ray diffraction and scanning electron microscopy, respectively.

The dielectric characterization of the obtained samples was performed in the radiofrequency range from 100 Hz to 1 MHz and in the microwave range at 2.7 GHz using the impedance spectroscopy technique and the resonant cavity method, respectively. 

## 2. Materials and Methods

### 2.1. Synthesis

In this work, the obtained powders were prepared via two different procedures, both based on the sol-gel method: the colloidal gel method and the polymeric gel method.

For the colloidal gel method, stoichiometric amounts of the precursors NbCl_5_ (Merck (Kenilworth, NJ, USA), >98%) and FeCl_3_ (Riedel-de Haën (Charlotte, NC, USA), >98%) were used. The FeCl_3_ was added to the NbCl_5_, which was previously dispersed in deionized water. The mixture was vigorously stirred at 60 °C until a gel was formed. To adjust the pH to 2.4, NH_4_OH (Alfa Aesar (Haverhill, MA, USA)) was added. The mixture was calcined at 300 °C for 6 h. To establish the heat-treatment temperatures, the obtained powders were subject to thermal measurements, which were carried out with Hitachi STA7300 equipment (Hitachi, Woodland, CA, USA) in a nitrogen atmosphere in a temperature range from room temperature to 1100 °C with a heating rate of 5 °C/min.

For the polymeric gel method, stoichiometric amounts of the precursors NbCl_5_ (Merck, >98%) and Fe(C_5_H_7_O_2_)_3_ (Aldrich (St. Louis, MO, USA), >97%) were used. The NbCl_5_ was previously dissolved in ethanol and stirred at 70 °C for 1 h. The Fe(C_5_H_7_O_2_)_3_ was added and the stirring was continued at 70 °C for 3 h, and the obtained mixture was calcined at 300 °C for 6 h. The obtained powder was subject to the same thermal measurements.

**Figure 3:**
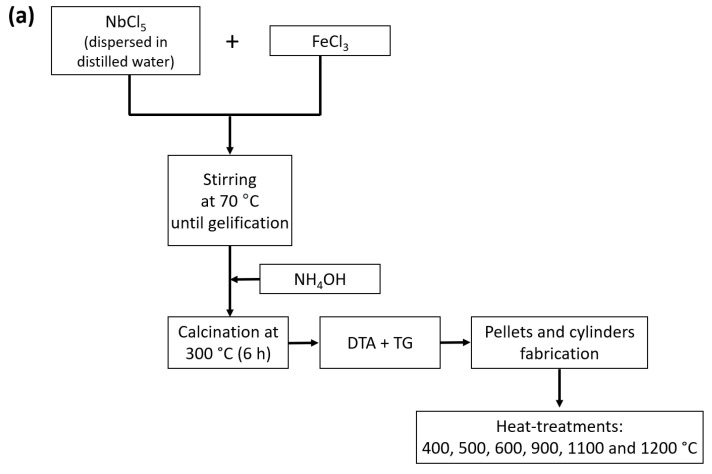

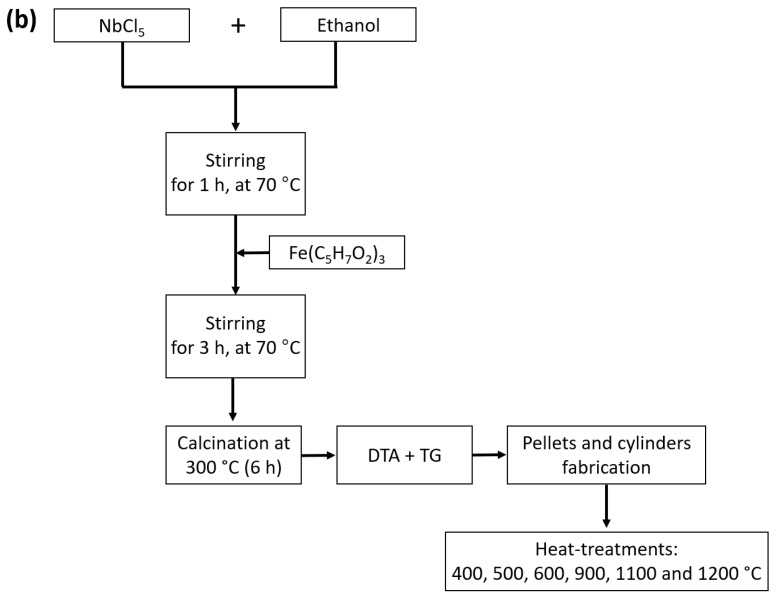
Flow chart for the (**a**) colloidal gel and (**b**) polymeric gel methods.

Figure 3a,b show the flow chart for the preparation of the samples via the colloidal gel and the polymeric gel methods, respectively. These diagrams include the heat-treatment temperatures, which were performed in a conventional electric furnace using a dwell time of 4 h and a heating rate of 5 °C/min. Before the heat-treatment process, the obtained powders were pressed into pellets and cylinders (5–7 mm in diameter) due to the requirements of the electrical measurement techniques. 

**Figure 4:**
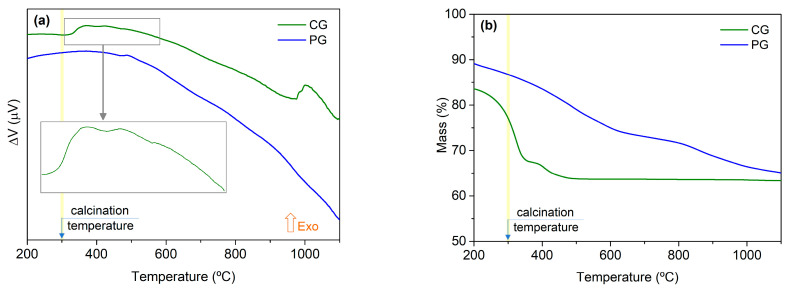
Thermograms of the obtained powders: (**a**) DTA; (**b**) TGA.

Figure 4a shows the results from the differential thermal analysis (DTA) for both methods, allowing for the evaluation of the temperatures at which crystalline phases can be formed. Only exothermic phenomena were identified, and these phenomena were more pronounced in the powder prepared via the colloidal gel method. Besides this, the thermogram representing the colloidal gel method suggested that for temperatures above 1100 °C, an exothermic phenomenon should probably exist. Based on these results, the selected temperatures for the heat treatments in the powders obtained from both methods were governed by the colloidal gel method thermogram, with temperatures between 400 and 1200 °C.

Figure 4b shows depicted the results from the thermogravimetric analysis (TGA), where one can see that in the powders prepared via the colloidal gel method, the weight loss stabilized at 500 °C. In the powders prepared via the polymeric gel method, the weight loss was smoother but extended to at least 1100 °C. 

The thermal measurements were carried out with Hitachi STA7300 equipment in a nitrogen atmosphere in a temperature range from room temperature up to 1100 °C with a heating rate of 5 °C/min.

Hereafter, the preparation methods, namely, colloidal gel and polymeric gel, are designated as CG and PG, respectively, and the samples are designated as HT followed by the corresponding treatment temperature, e.g., HT 400 for the sample treated at 400 °C.

### 2.2. Characterization

The X-ray diffraction (XRD) data were collected in an Aeris-PanAnalytical diffractometer (CuKα radiation, λ = 1.54060 Å) at 15 kV and 30 mA with a curved graphite monochromator, an automatic divergence slit, a progressive receiving slit and a flat plane sample holder in a Bragg–Brentano parafocusing optics configuration. Intensity data were collected in the 2θ angle range of 10°–60° via the step counting method (step 0.02° in 1 s). To confirm the crystal structure and acquire supplementary structural information about the samples, Rietveld refinement was carried out using Profex [22].

The morphologies of the samples were analyzed via scanning electron microscopy (SEM). The images were obtained on a TESCAN-Vega III instrument. Before the microscopic observation, the samples were covered with carbon.

The density measurements were performed via the Archimedes method using a RADWAG-As220.R2 analytical balance with the specific density measurement device for solid materials. The balance had a measuring accuracy of ±0.1 mg and the fluid used was ethanol. The measurements were made at room temperature (22 °C) and 10 times for each sample. After the measurement for each sample, a dry process was undertaken in an oven with forced circulation at 100 °C for 1 h. Then, and before any new measure, each sample was kept in a desiccator with silica gel for a period longer than 2 h to ensure the same sample temperature and dryness.

The electrical measurements in the frequency range from 100 Hz to 1 MHz were performed on the pellets using the precision impedance analyzer Agilent 4294A (Santa Clara, CA, USA) in the *C_p_*−*R_p_* configuration at room temperature. The electrodes were made by covering the opposite sides of the samples with silver conductive paste. The real (*ε*′) and imaginary (*ε*″) parts of the complex permittivity were obtained using the following relation:

(1) $$\varepsilon^{*}=\varepsilon^{\prime }-i\varepsilon^{\prime \prime }=C_{p}\frac{d}{A\varepsilon_{0}}-i\frac{d}{\omega R_{p}A\varepsilon_{0}}$$

where *C_p_* represents the capacitance, *R_p_* the resistance, *d* the sample thickness, *A* the electrode (pellet) area, *ε*_0_ the empty space permittivity and *ω* the angular frequency [23].

The measurements of the complex permittivity in the microwave range were made at ≈2.8 GHz at room temperature in a resonant cavity operating in the TE_105_ mode and coupled to an HP 8753D Network Analyzer. When the sample was positioned in the center of the cavity, where the electric field was maximal, the variation in the resonant frequency Δ*f* and the inverse of the quality factor Δ(1/*Q*) of the resonant cavity could be correlated to the permittivity values [24]. The real part of the complex permittivity was obtained through the shift in the resonant frequency of the cavity and the imaginary part using the change in the inverse of the quality factor of the cavity. Applying the small perturbation theory and considering only the first-order perturbation in the electric field caused by the sample [24,25], *ε*′ and *ε*″ could be calculated using Equation (2):

(2) $$\varepsilon^{*}=\varepsilon^{\prime }-i\varepsilon^{\prime \prime }=\left(K\frac{\Delta f}{f_{0}}\frac{V}{v}+1\right)-i\left(\frac{K}{2}\Delta \left(\frac{1}{Q}\right)\frac{V}{v}\right)$$

where *K* is a constant related to the depolarization factor that depends on geometric parameters; *V* and *v* are the volumes of the cavity and the cylindrical sample, respectively; and *f*_0_ is the resonance frequency of the cavity. Using a sample with a known dielectric constant, commonly polytetrafluorethylene (PTFE), with the same shape and dimensions as the ceramic samples, the constant *K* could be determined and, consequently, *ε*′ and *ε*″ could be calculated.

## 3. Results

### 3.1. Structural Characterization

**Figure 5:**
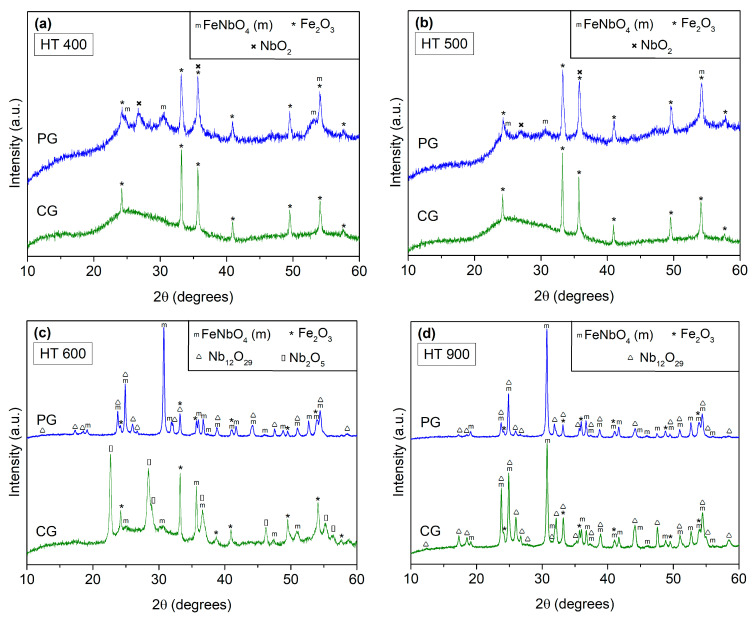

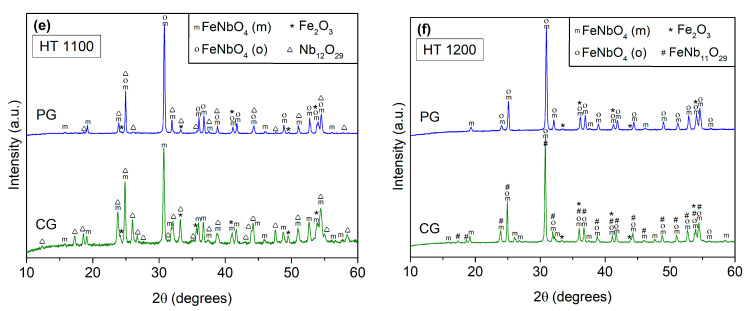
X-ray diffraction patterns of the prepared samples: (**a**) HT 400; (**b**) HT 500; (**c**) HT 600; (**d**) HT 900; (**e**) HT 1100; (**f**) HT 1200.

The crystalline phases were identified via XRD, as shown in Figure 5a–f.

**Figure 6:**
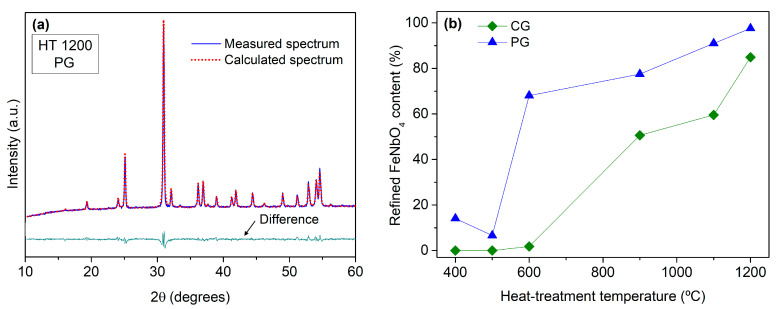
(**a**) Measured and calculated spectrum of the sample treated at 1200 °C and prepared via the polymeric gel method; (**b**) variation percentage of the FeNbO_4_ content as a function of the heat-treatment temperature, as estimated via the Rietveld analysis.

In the heat treatments performed at 400 and 500 °C, Figure 5a and Figure 6b show that regardless of the preparation method, the presence of amorphous material was noticeable. However, in the powders prepared via the PG method, besides the secondary phases Fe_2_O_3_ and NbO_2_, monoclinic FeNbO_4_ (m-FeNbO_4_) was identified. With the CG method, the only crystalline phase present was the Fe_2_O_3_. In the samples treated at 600 °C with the PG method, besides the phases previously obtained, a new phase, namely, Nb_12_O_29_, was formed. In the sample prepared via the CG method, two new phases were formed: the Nb_2_O_5_ and the desired monoclinic FeNbO_4_. 

Increasing the treatment temperature to 900 °C, both samples showed the same composition: monoclinic FeNbO_4_ and the secondary phases Fe_2_O_3_ and Nb_12_O_29_.

Considering the heat treatment at 1100 °C, the sample prepared via the CG method maintained the three phases previously identified. However, with the PG method, besides these phases, the diffraction pattern showed peaks consistent with orthorhombic FeNbO_4_ (o-FeNbO_4_).

Finally, with the heat treatment at 1200 °C, the polymorphs m-FeNbO_4_ and o-FeNbO_4_ were present in both samples, along with the secondary phase Fe_2_O_3_. The CG method promoted the formation of an additional phase, namely, FeNb_11_O_29_.

Comparing these preparation procedures, one can assume that with the PG route, the monoclinic and the orthorhombic FeNbO_4_ can be formed at lower temperatures when compared with the CG method. Even so, the result achieved with both methods, that is, the temperature of transition between monoclinic and orthorhombic structure, was aligned with the literature. 

The content of each crystalline phase identified in the prepared samples was estimated via Rietveld’s refinement method using Profex [22]. Figure 6a shows the measured and calculated spectra for the HT 1200 sample prepared via the PG method. Figure 6b presents the evolution of the percentage of the FeNbO_4_ content with the increase in the heat-treatment temperature. Besides the earlier formation of FeNbO_4_ with the PG method already mentioned, this graphic also shows that regardless of the treatment temperature, the content of FeNbO_4_ was always higher in the samples prepared via the PG route. 

Table 1 displays the percentage of each crystalline phase identified in the samples and also the Rietveld fitting parameters, which show the good quality of the fitting [26] and the consistency of the presented results.

As many of the authors mentioned in the Introduction section, single-phase m-FeNbO_4_ or o-FeNbO_4_ was not achieved. However, with the PG method and a 4 h heat treatment at 1200 °C, only FeNbO_4_ and the starting material Fe_2_O_3_, with a content inferior to 3%, were identified.

This structural characterization shows that contrary to what was previously reported, it is possible to obtain m-FeNbO_4_ using the CG and PG methods. Furthermore, contrasting with the literature, the secondary phases FeNb_2_O_6_ and Fe_4_Nb_2_O_9_ were not identified. 

The development of materials that can be obtained with lower sintering temperatures in a reduced processing time is also advantageous. In our previous work [18], pure m-FeNbO_4_ was obtained via the sol-gel method but with a stirring time of 7 days. Additionally, in the referenced works where wet chemical methods were applied, elevated stirring and heat-treatment times were adopted. 

### 3.2. Morphological Characterization

**Figure 7:**
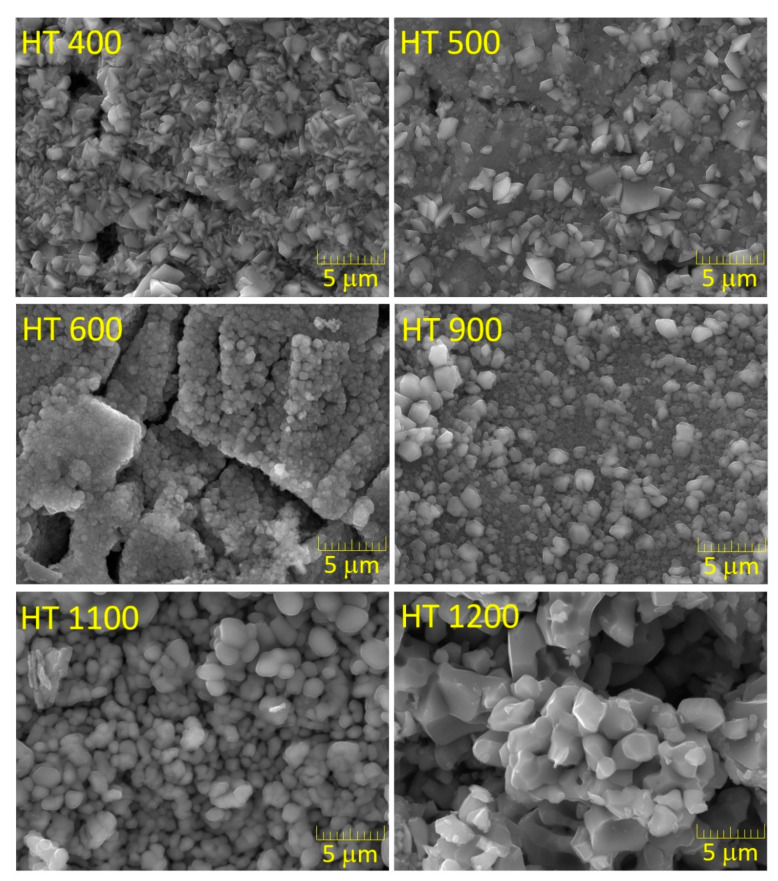
SEM micrographs of the samples prepared via the colloidal gel method.

Figure 7 shows the SEM micrographs of the samples prepared via the CG method. 

The HT 400 and HT 500 samples were composed of particles with an angular shape and significant heterogeneity in terms of size. The heat treatment at 600 °C promoted a substantial change in the morphology of the material since both the shape and size of the grains were very similar in this sample. This evolution could have been due to the conversion of the amorphous material and the formation of new crystalline phases. With the heat treatment at 900 °C, the grain growth was visible, which is a factor that contributed to the heterogeneity of the sample. With the increase in the treatment temperature, the particles became progressively bigger, with this increase being more pronounced in the HT 1200 sample, possibly due to the decrease in the Fe_2_O_3_ content. 

**Figure 8:**
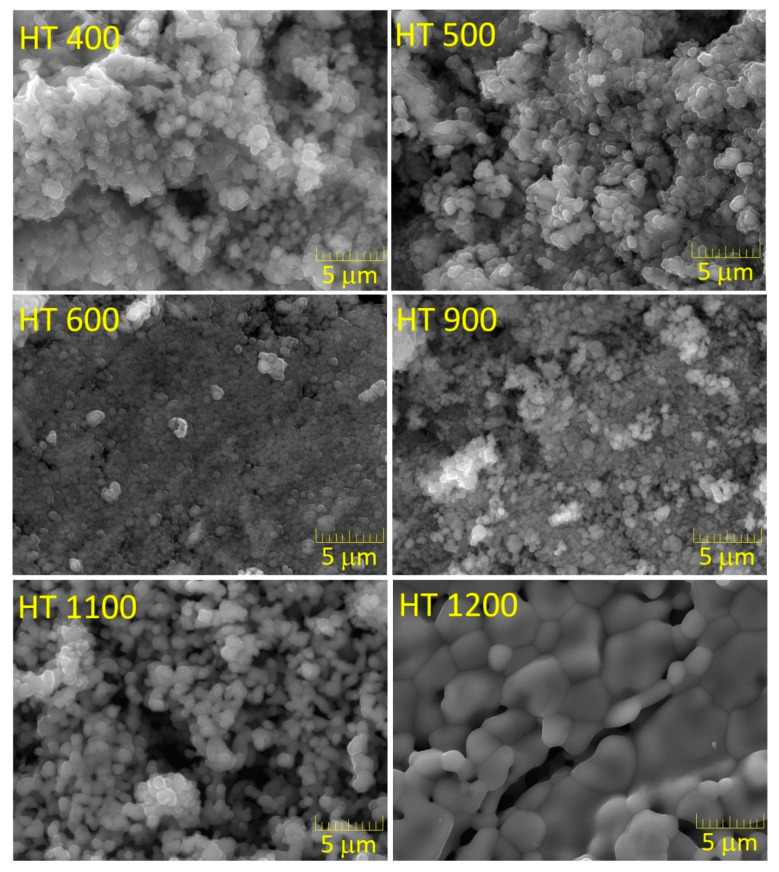
SEM micrographs of the samples prepared via the polymeric gel method.

Figure 8 shows the SEM micrographs of the samples prepared via the PG method. 

The morphologies of the HT 400, HT 500, HT 600 and HT 900 samples were similar. The slight decrease in the particle size promoted by the heat treatment at 600 °C could be due to the decomposition of the amorphous material. 

As seen in the case of the CG method, the increase in the treatment temperature promoted grain growth; however, these changes were much more distinctive in the HT 1200 sample, where the grains could be greater than 5 μm, with the grain’s boundary being very well defined. 

**Figure 9:**
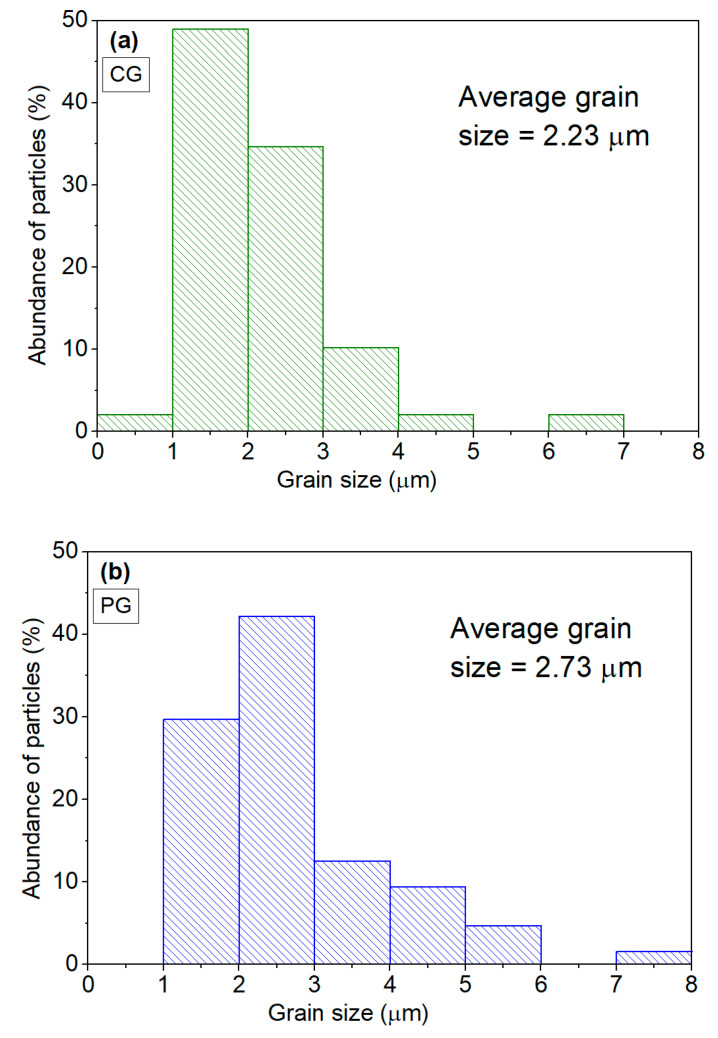
Grain size distribution of the HT 1200 sample prepared via the (**a**) colloidal gel and (**b**) polymeric gel methods.

The grain size distributions for the HT 1200 samples prepared via both methods are shown in Figure 9. The data collection was performed using two SEM images to improve the statistical calculation [27]. In the sample prepared via the CG method, the average grain size was 2.23 μm, with only 2% of the analyzed grains being greater than 5 μm. For the sample obtained via the PG method, the average grain size was 2.73 μm, with 6.25% of the analyzed grains being greater than 5 μm.

Despite the structural differences, the grain size distribution and, consequently, the average grain size were very similar in both samples.

### 3.3. Density Measurements

Table 2 shows the experimental densities of the HT 400 to HT 1100 samples. In general, the heat treatment promoted the densification of the samples. Moreover, independent of the treatment temperature, the density of the samples prepared via the PG method was always higher. This fact can be explained by the higher content of m-FeNbO_4_, which presents a higher theoretical density than the secondary phases. 

Considering the samples with higher amounts of m-FeNbO_4_, namely, HT 1100 for the CG method and HT 900 for the PG method, the calculations showed that the experimental density was 81.3% of the theoretical density in both cases, with the samples presenting a theoretical density of 5.14 and 5.24 g/cm^3^, respectively. 

The theoretical density was obtained using the following equation [28]

(3) ρth=ω1+ω2+ω3ω1ρ1+ω2ρ2+ω3ρ3 

where *ω*_1_, *ω*_2_ and *ω*_3_ are the mass fractions presented in Table 1, and *ρ*_1_, *ρ*_2_ and *ρ*_3_ are the theoretical densities of Fe_2_O_3_ (5.27 g/cm^3^), m-FeNbO_4_ (5.38 g/cm^3^) and Nb_12_O_29_ (4.57 g/cm^3^), according to the Pearson’s Crystal Data database [29] and the references [8,30,31], respectively.

The good densification of the samples was associated with a low porosity, which is a physical characteristic that can be related to lower dielectric losses [32]. 

### 3.4. Dielectric Characterization

**Figure 10:**
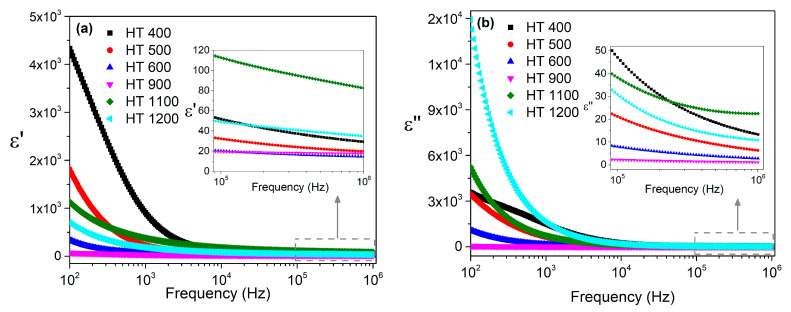
Frequency dependence of the (**a**) dielectric constants and (**b**) dielectric losses at room temperature for the samples prepared via the colloidal gel method.

The dielectric constants *ε*′ and the dielectric losses *ε*″ of the samples prepared by the colloidal gel method, as measured at room temperature and in the frequency range of 100 Hz to 1 MHz, are shown in Figure 10. 

In the low-frequency region, the dielectric constant decreased sharply with the frequency, with the HT 400 sample showing higher values. In the high-frequency region, the *ε*′ values decreased smoothly, with the HT 1100 sample standing out.

The dielectric losses presented a similar trend; however, in the low-frequency region, the sample with the higher grain size, i.e., HT 1200, presented higher *ε*″ values. 

**Figure 11:**
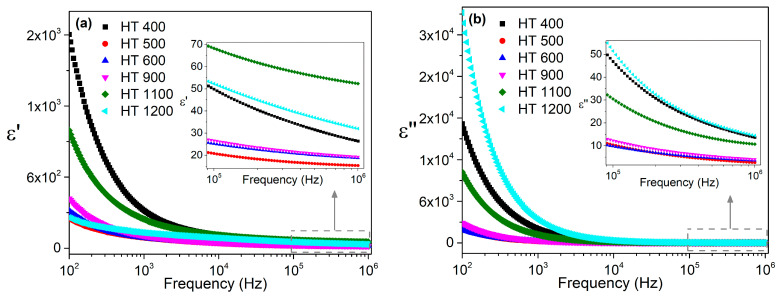
Frequency dependence of the (**a**) dielectric constants and (**b**) dielectric losses at room temperature for the samples prepared via the polymeric gel method.

The dielectric constants *ε*′ and the dielectric losses *ε*″ of the samples prepared by the polymeric gel method, as measured at room temperature and in the frequency range of 100 Hz to 1 MHz, are presented in Figure 11. The abrupt decrease in the low-frequency region, followed by a smooth decrease for higher values of frequency, of *ε*′ and *ε*″ was similar to the behavior of the samples prepared via the colloidal method. The dielectric constants and losses obtained for the samples prepared via the two methods were of the same order of magnitude.

Comparing the present results with pure m-FeNbO_4_ prepared via the sol-gel method [18], one can see lower values for the dielectric constant; however, the losses were also several orders of magnitude lower. These differences can be attributed to the existence of secondary phases, but also due to the less successful densification of the samples. 

In addition, the dissipation factor or loss tangent tanΔ is often used to characterize the dielectric loss of a material and is given by [33]

(4) $$\tan\delta =\frac{\varepsilon^{\prime \prime }}{\varepsilon^{\prime }}$$

The real part of any dielectric function varies monotonically with frequency, whereas its imaginary part may display a maximum as a function of frequency. However, the loss tangent behaves like an imaginary part. Whenever a maximum occurs, the peak location can be represented by the relaxation time, which is defined as the inverse angular frequency at the maximum of the imaginary part [33].

**Figure 12:**
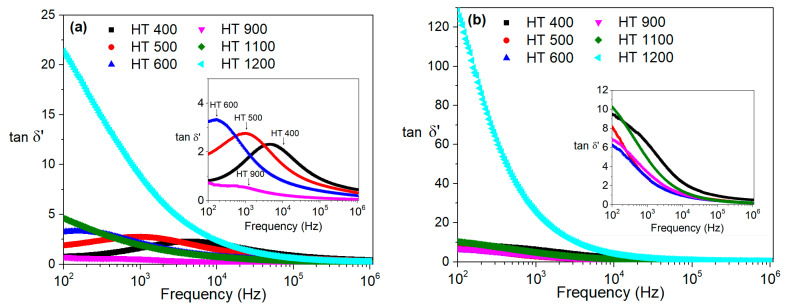
Loss tangent at room temperature for the samples prepared via the (**a**) colloidal gel and (**b**) polymeric gel methods.

After analyzing the loss tangent for the studied samples, with the results depicted in Figure 12, it was possible to conclude that the HT 400, HT 500 and HT 600 samples prepared via the CG method had a relaxation phenomenon at room temperature in the frequency range used. The increase in the treatment temperature was followed by a decrease in the frequency of the peak, which means that the increase in the treatment temperature culminated in an increase in the relaxation time. 

Since these relaxation phenomena were not perceptible with the complex permittivity formalism, other approaches were used.

Several dielectric functions can be used to describe the frequency-dependent properties of a material. Among them, the most commonly applied are the complex dielectric constant (ε*), complex electric modulus (M*), complex impedance (Z*) and complex admittance (Y*) [34].

The real and imaginary parts of the electric modulus are related to the permittivity via the following equations [35]:

(5) $$M^{\prime }=\frac{\varepsilon^{\prime }}{{\varepsilon^{\prime }}^{2}+{\varepsilon^{\prime \prime }}^{2}}$$

(6) $$M^{\prime \prime }=\frac{\varepsilon^{\prime \prime }}{{\varepsilon^{\prime }}^{2}+{\varepsilon^{\prime \prime }}^{2}}$$

where *M*′ and *M*″ are the real and imaginary parts of the complex modulus.

One of the benefits of employing the modulus formalism to study the dielectric relaxation phenomena is the fact that the large variations in permittivity and conductivity that occur at low frequencies are minimized. Consequently, the recurrent constraints of the electrode nature and contact, space charge injection phenomena and absorbed impurity conduction effects, which could obscure the relaxation mechanism in the permittivity representation, can be overcome. Moreover, the contribution of electrode polarization effects on the modulus data can be reduced, ensuring good ohmic contact between the electrodes and the sample, namely, through the use of silver paint contacts [36,37].

The real and imaginary parts of the impedance can be obtained from the following relations:

(7) $$Z^{\prime }=\frac{\varepsilon^{\prime \prime }d}{\omega \varepsilon_{0}A\left({\varepsilon^{\prime }}^{2}+{\varepsilon^{\prime \prime }}^{2}\right)}$$

(8) $$Z^{\prime \prime }=\frac{\varepsilon^{\prime }d}{\omega \varepsilon_{0}A\left({\varepsilon^{\prime }}^{2}+{\varepsilon^{\prime \prime }}^{2}\right)}$$

With the application of different formalisms, different relaxation times are obtained; however, all of them describe the same relaxation process, but from different points of view [33].

**Figure 13:**
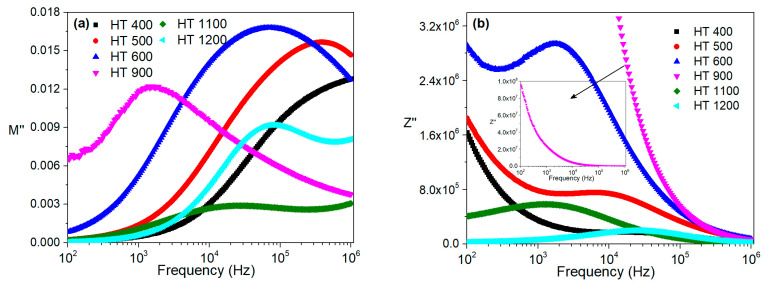
Frequency dependence of the imaginary parts of the (**a**) modulus and (**b**) impedance at room temperature for the samples prepared via the colloidal gel method.

**Figure 14:**
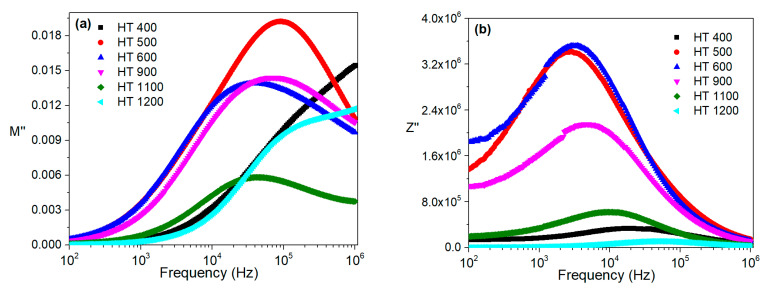
Frequency dependence of the imaginary parts of the (**a**) modulus and (**b**) impedance at room temperature for the samples prepared via the polymeric gel method.

Figure 13 and Figure 14 show the imaginary parts of the modulus and impedance of the samples prepared by both methods at room temperature and in the frequency range of 100 Hz to 1 MHz. 

Regarding the modulus formalism, for the CG method (Figure 13a), one can infer that the HT 400 sample had a relaxation mechanism, with the peak maximum occurring at a frequency higher than 1 MHz. The remaining samples had relaxation phenomena that were clearly visible in the analyzed frequency range. With the impedance formalism (Figure 13b), the relaxation mechanism of the HT 900 sample was not visible. 

For the PG method, the modulus representation, which is depicted in Figure 14a, shows that all the samples, except for HT 400, had a relaxation process between 100 Hz and 1 MHz. This observation was confirmed by the impedance formalism (Figure 14b), where all the samples had one peak.

**Figure 15:**
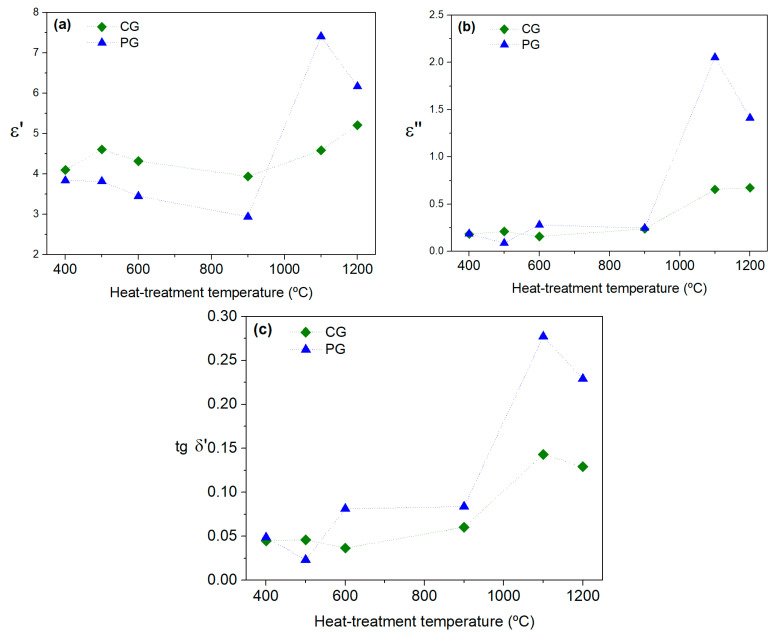
(**a**) Dielectric constants, (**b**) dielectric losses and (**c**) loss tangents at room temperature for the samples prepared via the colloidal gel and polymeric gel methods.

Figure 15 shows the dielectric constant, the dielectric losses and the loss tangent obtained at room temperature at ≈2.7 GHz. The dielectric constant and dielectric losses showed the same trend, regardless of the preparation method. As was observed for the radiofrequency range measurements, in the microwave region, the order of magnitude of the calculated *ε*′ and *ε*″ were the same for both methods. 

The loss tangent was generally higher for the samples prepared via the PG method and increased with the increase in the treatment temperature. However, both methods generated samples with tanΔ smaller than one.

## 4. Conclusions

Monoclinic and orthorhombic iron niobate were successfully prepared via the CG and the PG methods, which is an achievement that contrasts with the literature since with these methods, only the formation of o-FeNbO_4_ was reported. 

For the PG method, the m-FeNbO_4_ was already present in the sample that was heat-treated at 400 °C, with the conversion into o-FeNbO_4_ starting at 1100 °C. For the CG method, the formation of m-FeNbO_4_ and o-FeNbO_4_ occurred at 400 °C and 1200 °C, respectively. 

Considering the powders that were treated at the higher temperature, the sample from the PG method had a combination of monoclinic and orthorhombic FeNbO_4_, as well as a secondary phase with a content smaller than 5%. The CG method produced a sample also with m-FeNbO_4_ and o-FeNbO_4_, but with two secondary phases totaling about 15% of its content. Taking into consideration the previous statements and the fact that the processing procedures had similar stages, it is possible to conclude that the PG method has higher potential.

The heat treatment promoted the densification of the samples, with both methods showing promising results. 

The dielectric characterization of the HT 1100 and HT 1200 samples showed that the increase in the o-FeNbO_4_ content may have contributed to the decrease in the dielectric constants and the increase in the losses. The samples from both methods started by benefiting from the formation of this polymorph; however, the increase in its content had a negative effect, which was visible in the HT 1200 sample created using the PG method. In this sample, the increase in the o-FeNbO_4_ coincided with the decrease in the dielectric constant and the increase in the dielectric loss, with this last effect showing an attenuation with the increase in the frequency.

Considering the room temperature dielectric properties of m-FeNbO_4_ prepared via the sol-gel method, the samples prepared via the GG and PG methods presented losses that were also several orders of magnitude lower, which can be considered a promising result. 

At room temperature, in the frequency range analyzed, all the samples showed one relaxation mechanism.

## Figures and Tables

**Table 1:** Crystalline phases identification and percentages estimated via the Rietveld analysis for the prepared samples, along with the Rietveld fitting parameters.

Heat-Treatment Temperature	Colloidal Gel Method				Polymeric Gel Method			
	Crystalline Phases	Rietveld Fitting Parameters			Crystalline Phases	Rietveld Fitting Parameters		
		R_wp_	R_exp_	χ^2^		R_wp_	R_exp_	χ^2^
HT 400	Fe_2_O_3_—100%	2.49	1.51	2.71	Fe_2_O_3_—77.50% m-FeNbO_4_—14.10% NbO_2_—8.40%	2.70	1.64	2.71
HT 500	Fe_2_O_3_—100%	2.52	1.47	3.03	Fe_2_O_3_—91.99% m-FeNbO_4_—6.61% NbO_2_—1.40%	2.38	1.54	3.03
HT 600	Fe_2_O_3_—9.10% m-FeNbO_4_—1.80% Nb_2_O_5_—89.10%	4.05	1.47	7.59	Fe_2_O_3_—13.78% m-FeNbO_4_—68.06% Nb_12_O_29_—18.16%	2.26	1.30	3.02
HT 900	Fe_2_O_3_—10.05% m-FeNbO_4_—50.64% Nb_12_O_29_—39.31%	3.12	1.40	4.97	Fe_2_O_3_—8.79% m-FeNbO_4_—77.52% Nb_12_O_29_—13.69%	2.08	1.38	2.27
HT 1100	Fe_2_O_3_—9.85% m-FeNbO_4_—59.58% Nb_12_O_29_—30.57%	2.76	1.64	2.83	Fe_2_O_3_—4.58% m-FeNbO_4_—72.89% o-FeNbO_4_—18.14% Nb_12_O_29_—4.39%	2.06	1.35	2.33
HT 1200	Fe_2_O_3_—2.75% m-FeNbO_4_—61.00% o-FeNbO_4_—23.84% FeNb_11_O_29_—12.41%	2.07	1.43	2.10	Fe_2_O_3_—2.40% m-FeNbO_4_—61.00% o-FeNbO_4_—36.60%	2.44	1.51	2.61

**Table 2:** Measured densities of the samples prepared via the colloidal gel and polymeric gel methods.

Heat-Treatment Temperature	Colloidal Gel Method (g/cm^3^)	Polymeric Gel Method (g/cm^3^)
HT 400	3.352 ± 0.102	3.887 ± 0.106
HT 500	3.682 ± 0.025	4.051 ± 0.038
HT 600	4.090 ± 0.105	4.185 ± 0.055
HT 900	4.014 ± 0.288	4.261 ± 0.235
HT 1100	4.180 ± 0.019	4.881 ± 0.115

## Data Availability

The raw/processed data required to reproduce these findings cannot be shared at this time, as the data also form part of an ongoing study.
